# Potential Pitfalls in Wastewater Phosphorus Analysis and How to Avoid Them

**DOI:** 10.1177/11786302211019218

**Published:** 2021-05-30

**Authors:** Praveen Rosario, Ramya Viswash, Thamayanthi Seenivasan, Sudha Ramalingam, Katelyn L Sellgren, Sonia Grego, Lena Trotochaud

**Affiliations:** 1 RTI International India, New Delhi, India; 2 PSG Institute of Medical Sciences and Research, Coimbatore, TN, India; 3 Center for Water, Sanitation, Hygiene, and Infectious Disease (WaSH-AID), Department of Electrical and Computer Engineering, Duke University, Durham, NC, USA

**Keywords:** Wastewater, nutrient pollution, standard solutions, onsite treatment, phosphorus, phosphate, non-sewered sanitation

## Abstract

Due to the increasing adoption of nutrient discharge regulations, many research groups are stepping into new territory with phosphorus (P) measurements. Accurate reporting of P concentrations in effluent from novel wastewater treatment technologies is critical for protecting both environmental and human health. Analysis of P in wastewater is prone to pitfalls because of the (1) variety of chemical forms of P in wastewater (orthophosphate, condensed P, and organic P), (2) availability of different chemical assays for measuring different P forms, and (3) different conventions in the units for reporting P. Here, we present a case study highlighting how these pitfalls affect analysis and interpretation of P measurements. We show that, when used appropriately, commercially-available kits are indeed accurate tools for evaluating reactive P and total P concentrations. For both standard solutions and real wastewater, we systematically remove steps from the total P protocol to show how protocol deviations affect the results. While standard solutions are important for validating analytical methods, commercially-available wastewater standard solutions only contain P as orthophosphate (reactive P). We therefore demonstrate options for making a mixed-P standard solution containing acid-hydrolyzable and/or organic P compounds that can be used to validate both reactive P and total P assays.

## Introduction

An intense area of research is non-sewered sanitation solutions for rural areas and low- and middle-income countries^1-14^ to address the urgent need for safely-managed sanitation to improve human health and the environment.^15,16^ In addition to removing pathogens and organic compounds, a key requirement of wastewater treatment is removal of nutrients including nitrogen and phosphorus (N and P) due to their potential toxic effects on aquatic environments.^17,18^ The concern with P is that phosphates are limiting nutrients in freshwater aquatic environments; an overabundance can lead to decreased dissolved oxygen, accelerated eutrophication, and harmful algal blooms.^19^ These algal blooms can also pose a human health risk through the production of cyanotoxins.^20^ Additionally, P is a limited, valuable natural resource that should be recovered and reused.^21,22^

Nutrient discharges from inadequate onsite wastewater treatment systems are a growing concern globally and likely underreported.^16,23^ Affordable and sustainable methods for effective N and P removal in decentralized treatment systems remain technologically challenging.^24,25^ Recently, an international standard ISO 30500 was specifically developed to provide performance requirements for non-sewered sanitation systems^26^ and sets a minimum threshold reduction of 80% for total P. As P regulations are increasingly enforced, wastewater P content will need to be regularly measured and reported as new technologies are pilot-tested.^2,14,27,28^ Standardized P reporting is required to compare performance across different technologies and wastewater streams to accelerate technology adoption, yet a critical gap exists due to lack of a common set of metrics.^29^ This knowledge gap in reporting metrics for P and its different chemical species is also highlighted in a recent systematic review of nutrient removal and recovery technologies by Kogler et al.^30^ In reviewing 292 articles across 46 sources, Kogler et al.^30^ found that removal efficiency and achievable effluent concentrations for P removal and recovery technologies are generally underreported, with only 16% of the articles reviewed giving adequate quantitative information for meaningful interpretation of technology performance.

As we embarked on the journey of adding total P to our routine testing regimen, we identified several specific points of confusion. We determined that measurement and reporting of P requires particular attention because of the (1) variety of chemical forms of P in wastewater, (2) availability of several different chemical assays for measuring different forms of P, and (3) different conventions in the units used for reporting P. Due to the interrelated nature of these topics, there is potential for inconsistencies in reporting results for P. Here, we present a case study highlighting the importance of accurate and clear reporting of P units and strict adherence to chemical assay protocols for measuring P in wastewater samples. The objective of this paper is to share our experiences in identifying and overcoming these P-related pitfalls so that others may avoid these issues in their future work. The aims of this paper include (1) determining the root cause(s) of discrepancies in P results and (2) developing new standard solution mixtures to ensure accurate analytical method implementation and data reporting.

We begin with a brief review of the forms of P in wastewater and typical units used for reporting P. We then describe our recent experiences where we identified discrepancies in P concentrations we measured using a commercially-available test kit and those measured by a third-party lab for the same samples. Due to the transparent and productive relationship with the third party lab, we were able to identify two distinct sources of confusion as the likely reason for these discrepancies: (1) the factor of 3.066 difference in results reported as mg L^−1^ “PO_4_-P” versus mg L^−1^ “PO_4_^3−^”; and (2) the omission of the digestion heating step in the total P protocol. By systematically omitting steps from the total P chemical assay procedure, we show that skipping the digestion heating step results in a significant undercounting of total P, both for standard solutions and for real wastewater samples.

To avoid these issues in future studies, regularly testing known P standard solutions to validate analytical methods and reporting methods is needed. However, we observed that the common commercially-available wastewater standard solutions only contain P as orthophosphate (reactive P). This is perhaps due to the fact that soluble orthophosphate is the majority P form in effluents from traditional wastewater treatment systems and therefore also the P form of primary concern for anthropogenic nutrient pollution.^31^ However, our analysis reveals that the assumption that orthophosphate = total P does not necessarily hold for wastewater influent of novel onsite wastewater treatment technologies. We therefore believe that including acid-hydrolyzable P and/or organic P components in known standard solutions is especially important to ensure accurate reporting of novel wastewater treatment technology performance. To this end, we have prepared and validated our own standard mixtures of orthophosphate, acid-hydrolyzable P, and organic P using readily available and inexpensive chemical reagents.

## Methods

Unless otherwise indicated, all glass wares used in this study were cleaned by soaking in 1:1 HCl:water (v:v), rinsing thoroughly in distilled or ultrapure water, then filling with fresh distilled or ultrapure water and storing until needed. Mean and standard deviation values for replicate measurements were calculated using Excel. Data were plotted using OriginPro 2018.

### Standard solutions

A mixed-parameter quality control standard solution for wastewater was purchased (Hach, product LCA720) that features 10.0 mg L^−1^ PO_4_-P in addition to ammonia, nitrate, COD, and TOC. A wastewater influent inorganics quality control standard solution was also purchased (Hach, product #2833149) that contains 10 mg L^−1^ PO_4_^3−^ in addition to ammonia, nitrate, COD, sulfate, and TOC. (See Supplemental Material for details.)

The other standard solutions used to validate assay performance were prepared in-house. Phosphate stock standard solutions (PO_4_^3−^ = 10 and 25 mg L^−1^, labelled P 10 and P 25, respectively) were prepared by dissolving potassium dihydrogen phosphate (KH_2_PO_4_) in distilled water. Mixed standard solutions were prepared using KH_2_PO_4_, sodium trimetaphosphate (Na_3_P_3_O_9_), and adenosine 5’-triphosphate, disodium salt (ATP), all purchased from Sigma-Aldrich (see Supplemental Material for additional reagent information).

### Real wastewater samples

Effluent from a toilet was collected to serve as wastewater samples for evaluation. A women’s shared toilet facility at a privately-owned textile mill in Coimbatore, India (described in a previous study) was the sample collection site.^13^ The facility is used by 20 to 50 employees and features five toilet stalls equipped with Indian squat plates, each connected to cistern flush. The effluent of one of the toilets can be easily tapped into from the pipe and was diverted from its septic tank to a 200 L collection tank. After collecting all toilet effluent (containing flush water, urine, and feces) from 8 am to 5 pm local time, the wastewater was left to settle overnight. The supernatant was collected the morning after and then the contents of the tank were homogenized by with a macerator pump (Jabsco Model: 18950, 45 lpm). Both supernatant and macerated wastewater samples were analyzed with the commercially-available test kit on the same day.

### Commercially-available test kits (ascorbic acid method)

Total P (method 8190) and reactive P (method 8048) test kits were purchased from Hach and used with a Hach DR 900 colorimeter. Unless otherwise indicated, the test kits were used exactly as per the manufacturer’s instructions (see Supplemental Material for more detail). Both test kits use the ascorbic acid method, are USEPA accepted for reporting wastewater analyses, and the procedures follow USEPA and Standard Method 4500-P.^32^ Distilled (in India) or ultrapure 18.2 MΩ cm (in the US) water was used for dilution as necessary to bring the samples within the assay detection range. The total P test kit includes two powder pillows (potassium persulfate, PhosVer 3), a bottle of sodium hydroxide 1.54 N, and glass reaction vials containing a pre-measured sulfuric acid solution. A Hach DRB 200 digester heating block was used for the digestion step in the total P protocol. The reactive P test kit includes one powder pillow (PhosVer3) and either glass reaction vials containing a pre-measured volume of deionized water or a reusable glass sample cell (see Supplemental Material).

### Third-party laboratory measurement (stannous chloride method)

Samples sent to a NABL certified laboratory (T.STANES Coimbatore) were analyzed using the Indian Standard IS:3025 part 31^33^ stannous chloride method for the measurement of total P, a method essentially similar to Standard Method 4500-P.^32^ According to the IS:3025 part 31 stannous chloride method, samples are decolorized by shaking with activated carbon for 5 minutes, then filtered. A phenolphthalein indicator is added to the filtered sample, and if a pink color develops, the pink color is discharged through the addition of up to 5 drops of a strong acid solution. Finally, ammonium molybdate and stannous chloride reagents are added and allowed to react for 10 minutes. The sample is then quantified spectrophotometrically at 690 nm. Fresh standard solutions were prepared by the third-party lab to calibrate their instrument upon receipt of each set of samples. Distilled water was used for sample dilution when necessary to bring the sample within the assay detection range. Analytical results from the third-party lab were reported to us in units of “Phosphorus (mg/L).”

## Results and Discussion

### Forms of P relevant for wastewater analysis

In aqueous solutions relevant for wastewater treatment technologies, P exists in three main forms, commonly referred to as orthophosphate, condensed phosphate, and organic phosphate (Table 1).^32^ Phosphate anions can be detected colorimetrically, and chemical assays are commercially available for measuring only orthophosphate (sometimes called “reactive P”), “acid-hydrolyzable P” (orthophosphate + condensed phosphate), or “total P” (orthophosphate + condensed phosphate + organic phosphate). The specific chemical reactions responsible for the colorimetric responses have been described in detail elsewhere^32^ and should be included with each commercially-available assay. Briefly, orthophosphate anions form a colored complex with a transition metal under the chemical assay conditions.

**Table 1.** Common assays, with naming and unit conventions, for forms of P in wastewater analysis.^32^

Commercially-available chemical assays	“Orthophosphate” or “reactive P”	“Acid-hydrolyzable P”	“Total P” or “total phosphate”
Forms of P detected	Orthophosphate	Orthophosphate + condensed phosphate	Orthophosphate + condensed phosphate + organic phosphate
Digestion required?	No	Yes	Yes
Strong oxidant required?	No	No	Yes
Typical reported units (mg L^−1^)	PO_4_^3−^ or PO_4_-P	PO_4_^3−^ or PO_4_-P	PO_4_-P or P

*The color response for all the different assays is due to detection of orthophosphate.* Other forms of P (condensed phosphate and organic phosphate) must first be converted to orthophosphate before they can be detected. Condensed phosphates require a preliminary acid hydrolysis step at elevated temperatures before the colorimetric response can be observed. Some organic phosphates also require addition of a strong oxidant to the acid hydrolysis step. Orthophosphate and acid-hydrolyzable P are typically reported using units of either mg L^−1^ “PO_4_^3−^” (orthophosphate) or “PO_4_-P” (orthophosphate as P). Total P is typically reported as mg L^−1^ “P.”

### Potential Pitfall #1: Distinguishing and converting between the different units used for P reporting

Note that the “typical” reporting units in Table 1 are just a guideline. In our experience, these do not appear to be hard rules, and all three units are used for reporting all types of phosphorus data. *The units alone will not tell you which forms of phosphorus have been measured.* It is therefore important to understand what the different units mean and how they are interrelated. Some standards, such as the ISO 30500, are written as percent reduction requirements; in this case, the units in which P quantities are reported is not particularly important as long as it is internally consistent within any given study. However, P units must still be accurately and clearly reported in order to compare values across different studies and to nominal values for other discharge standards. Briefly, units of “PO_4_-P” and “P” are equivalent and therefore interchangeable. “PO_4_^3−^” units are calculated differently and a mathematical conversion must be performed to compare to the other units.

Converting between values reported in the three different units requires accounting for the mass of atoms which are counted in each unit. Units of PO_4_-P and P only count the 1 P atom (30.97 amu). Therefore, values reported in mg L^−1^ PO_4_-P will be identical to values reported in mg L^−1^ P and no conversion is required. In contrast, units reported in PO_4_^3−^ count all atoms in the phosphate anion—1 P atom (30.97 amu) and 4 O atoms (each 16.00 amu). Values reported in mg L^−1^ PO_4_^3−^ can be converted to P or PO_4_-P by dividing by 3.066, which is the stoichiometric ratio of the mass of the phosphate anion (1P + 4O = 30.97 + 4(16.00) = 94.97 amu) to that of a single P atom (30.97 amu). Similarly, values reported in units of P or PO_4_-P can be converted to units of PO_4_^3−^ by multiplying by 3.066:

Eq. (1) $$\begin{array}{l} 1.000\;\mathrm{mg}\;L^{-1}P\;=\;1.000\;\mathrm{mg}\;L^{-1}{\mathrm{PO}}_{4}–P\\ \;\;\;\;\;\;\;\;\;\;\;\;\;\;\;\;\;\;\;\;\;\;\;\;\;\;\;\;\;\;\;\;\;\;\;\;\;\;\;\;\;\;\;\;\;\;\;\;\;=\;3.066\;\mathrm{mg}\;L^{-1}{\mathrm{PO}}_{4}^{3-} \end{array}$$

As an example, consider the two wastewater (WW) quality control standard solutions, both purchased from Hach for use in this study. The first WW standard solution (1) is listed by the supplier as containing “10.0 mg L^−1^ PO_4_-P,” while the other (2) is listed as containing “10 mg L^−1^ PO_4_^3−^.” It is an easy mistake at first glance to think that the phosphate concentrations of these solutions are equivalent. However, when we convert WW standard solution (1) to PO_4_^3−^ units, we see that it actually contains 3 times as much phosphate: (10.0 × 3.066) = 30.7 mg L^−1^ PO_4_^3−^. To directly compare these wastewater standard solutions, we will hereafter label them as (1) WW 30 and (2) WW 10 to reflect the expected phosphate concentration in PO_4_^3−^ units.

**Figure 1.**
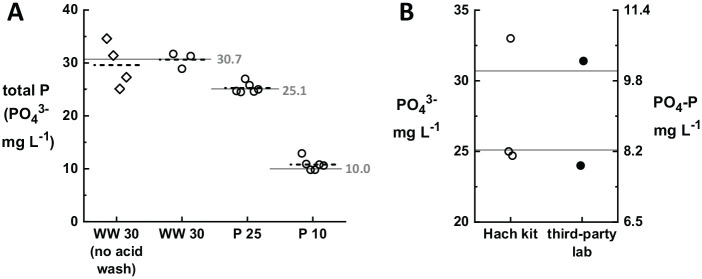
(A) Total P test results (measured by Hach kit) for the commercially-available standard solution WW 30 and in-house prepared phosphate solutions (P 25 and P 10). Each standard solution was tested multiple times; the results of individual tests are shown as open symbols, with the mean for each column shown as a short dashed line and the expected values shown as solid horizontal reference lines. The variability in test results is highest for WW 30 measured without acid washing of glassware (diamonds, n = 4, 29.6 ± 4.2 mg L^−1^ PO_4_^3−^). The variability is similar for all three standard solutions when glassware is acid washed prior to contact with the samples (circles; WW 30, n = 3, 30.6 ± 1.5; P 25, n = 6, 25.3 ± 1.0; P 10, n = 6, 10.8 ± 1.1). Position along the x-axis is not meaningful—data points within each column are offset from one another for clarity. (B) Total P measured for identical samples tested in-house with the Hach test kit and by the third-party lab. Values for units of PO_4_^3−^ are shown on the left y-axis and values for units of PO_4_-P are shown on the right y-axis. Expected values are shown as solid horizontal reference lines. Values reported by the third-party lab (in PO_4_-P units) are similar to those measured in-house (in PO_4_^3−^ units) once the correct unit conversion is applied.

We use a commercial test kit for total P (Hach 8190) for our in-house field testing, because this field-friendly method utilizes only two small, easily-transportable pieces of equipment (the DRB 200 digester and the DR 900 colorimeter) and pre-portioned reagents in pre-measured vials and sealed “powder pillow” packets. The results for total P are displayed by the DR 900 colorimeter in units of “mg L^−1^ PO_4_^3−^.” We used the WW 30 standard solution described above to validate the total P test kit, as recommended by the manufacturer. Results were obtained for samples collected in glass bottles cleaned with or without a HCl acid wash. (The acid wash is used to eliminate possible contamination from the detergent used to clean bottles, which may contain phosphate.) Figure 1 shows that all results obtained were within 15% of the expected value (n = 4; mean = 29.6 ± 4.2 mg L^−1^ PO_4_^3−^), however the variability in the acid-washed results was lower, within 5% of the expected value (n = 3; mean = 30.6 ± 1.5 mg L^−1^ PO_4_^3−^). We therefore used acid-washed containers for all subsequent measurements, including with samples sent for analysis to the third-party lab.

In addition to the WW 30 standard solution, we evaluated phosphate solutions prepared in-house of known concentrations (PO_4_^3−^ = 10, 25 mg L^−1^, labelled P 10 and P 25, respectively) obtained by dissolving KH_2_PO_4_ in distilled water. Again, these solutions gave results close to the expected values (P 10, n = 6, mean = 10.8 ± 1.1 mg L^−1^ PO_4_^3−^; P 25, n = 6, mean = 25.3 ± 1.0 mg L^−1^ PO_4_^3−^). These results confirm that solutions of KH_2_PO_4_ can effectively serve as standards for P in orthophosphate form, and that acid-washing of sample glassware reduces test-to-test variability in the results.

Initial reports showed different units for P analysis performed by a third-party lab, leading to a discrepancy which required further validation. Our third-party collaborator, a certified laboratory located in the same city, uses the stannous chloride method for total P measurement. This third-party lab requires a large volume (>150 mL) of sample to run their assay, thus we diluted by 5× the WW 30 standard solution prior to delivering it. We also sent for analysis a sample of the P 25 solution.

The third-party lab reported values of 10.25 mg L^−1^ for the WW 30 sample and 7.84 mg L^−1^ for the P 25 solution, both reported with units of “Phosphorus (mg/L).” Initially, we were surprised to find that the third-party lab results appeared much lower than the values we expected (30.7 and 25.1 mg L^−1^, respectively) for the WW 30 and P 25 samples. After clarifying discussions with the third-party lab, we determined that their results were reported in “PO_4_-P” units. Thus, the expected values would be 10.0 and 8.15 mg L^−1^ for units of PO_4_-P, respectively, for these two samples. In Figure 1B, the results are shown for both units of PO_4_^3−^ (on the left axis) and PO_4_-P (on the right axis). It is clearly seen that by resolving the ambiguity of the reported units, the total P values were measured by the third-party lab within a few percent from the expected values.

### Potential Pitfall #2: Omission of steps from the total P test protocols

Despite reconciling the difference in reported units between our in-house total P test results and those from the third-party lab, we noticed that there was still a discrepancy between results from the two labs when real wastewater samples were analyzed. Total P results from the third-party lab were always lower than those measured in-house, although not always lower by a consistent nominal amount or percentage. Importantly, we learned through fruitful discussions with the third-party lab that while a digestion (heating) pre-treatment step is listed in the IS:3025 protocol, digestion was not actually performed. This indicates that while the third-party lab results were accurate in determining total P content from the standard solutions (where only orthophosphate was present), their procedure will under-report the total P content for samples where condensed and organic phosphates are present (e.g., real wastewater samples from field testing evaluation of onsite treatment technologies).

**Figure 2.**
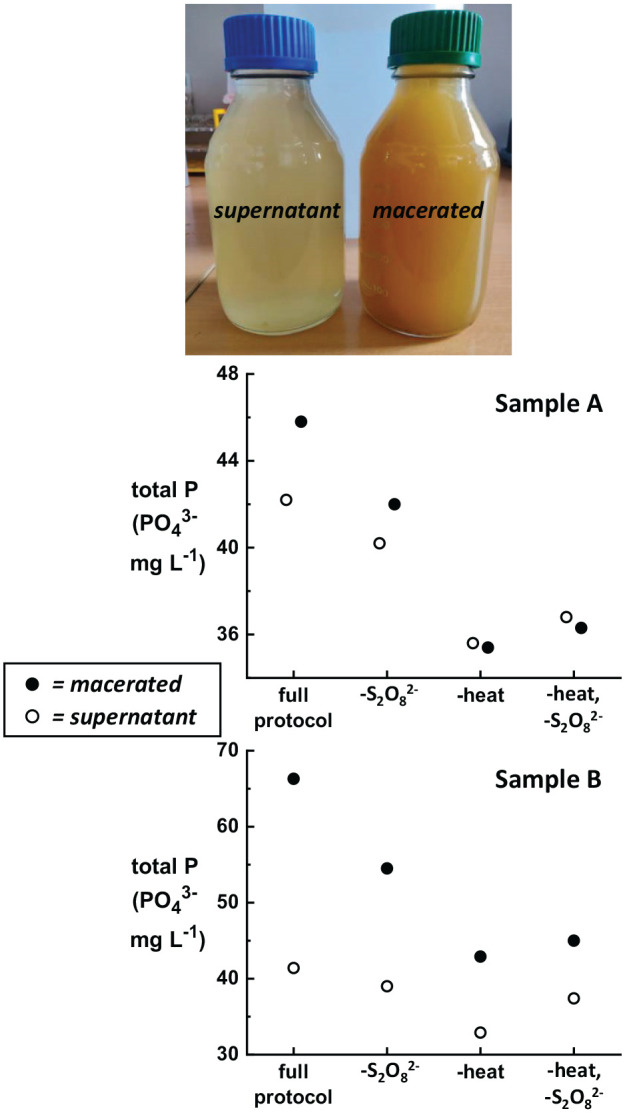
Comparison of total P results obtained during systematic omission of testing steps for two raw wastewater samples A and B collected from the Coimbatore field testing site. The photograph shows the typical appearance of the supernatant and macerated sample fractions. For each sample, both the macerated fraction (closed circles) and the supernatant fraction (open circles) were measured. The macerated fraction contains more fecal matter and thus a higher total P concentration. Omitting the heating step (“-heat”) from the testing protocol has the largest effect on the measured total P concentration, while omitting the persulfate reagent (“-S_2_O_8_^2−^”) has a smaller effect that is most visible for the macerated fraction. Samples were diluted with distilled water by 10× to 25× to bring them within the range of the total P Hach test kit.

The full protocol for Hach total P measurement includes a digestion step performed by heating the sample in acid at 105 °C for 30 minutes with a persulfate (S_2_O_8_^2−^) reagent added to the solution. Heating in acid converts any acid-hydrolyzable P to orthophosphate. The addition of the strong oxidant S_2_O_8_^2−^ ensures that organic phosphates are fully converted to orthophosphate. Figure 2 shows in-house results for two raw wastewater samples where steps from the total P protocol were systematically and intentionally omitted. Samples A and B were collected on different days from the same shared toilet effluent, and the liquid supernatant as well as a macerated sample containing higher fecal load were analyzed. The primary soluble form of P in wastewater is orthophosphate, therefore orthophosphate is expected to dominate in the supernatant fraction, while the macerated fraction is expected to contain a higher concentration of organic and condensed P forms.

Figure 2 shows that omitting the heating step has the largest effect on the total P results, both for the macerated and supernatant sample fractions. Omitting the S_2_O_8_^2−^ reagent has a measureable, but smaller, effect. When the protocol is followed fully, the total P measured for Sample A was 45.8 mg L^−1^ PO_4_^3−^ for the macerated fraction and 42.2 mg L^−1^ PO_4_^3−^ for the supernatant fraction. These values drop to 35.4 and 35.6 mg L^−1^ PO_4_^3−^, respectively, when the heating step is omitted. This results in an under-reporting of total P by 23% for the macerated fraction and 16% for the supernatant. For Sample B, omitting the heating step results in under-reporting of total P by 35% for the macerated fraction and 21% for the supernatant. Omitting only the S_2_O_8_^2−^ reagent under-reported the total P in the supernatant samples by ~5%, which is similar to the test-to-test variability for standard solutions as shown in Figure 1. In contrast, omitting only the S_2_O_8_^2−^ reagent from the macerated fraction under-reports the total P by 8% for Sample A and 18% for Sample B.

In summary, total P content in raw wastewater samples can be substantially under-reported if any step in the digestion protocol is omitted. The largest differences in total P values were found with heating and/or persulfate omission for the macerated sample fractions. This shows that the risk of P under-reporting increases as the fecal content (and thus the amount of condensed and/or organic P) increases. This is important when testing new treatment technologies, particularly those which involve novel solid/liquid separation processes, solids-settling techniques, or other methods of solids treatment where solid fecal matter is broken down and mixed with liquid effluent.

### Impact of the pitfalls

While the major contribution of water soluble P in human excreta is inorganic orthophosphate, orthophosphate may not always be the sole contributor to total P in effluent from onsite wastewater treatment technologies. Raw influent of onsite wastewater treatment technologies is highly variable and impacted by many factors such as number of toilet uses, flush volume, and diet.^34,35^ We suspect that concentrations of condensed and/or organic P may be significant in influent and in effluent from some technologies with novel solid/liquid separation or solids processing methods.

Our group has previously evaluated one such novel system. We reported in a previous publication total P values of system effluent of 8.3 and 21.5 “mg P L^−1^”^5^ that we now suspect were measured omitting the heating step; thereby, those values represent only the orthophosphate as opposed to the total P. In a different publication,^13^ we reported values of “P,” without specifying the form (which was orthophosphate) nor clarifying the reporting units (mg L^−1^ P). In another recent paper,^14^ we did specify in the methods section that both reactive P and total P were reported in units of “mg L^−1^ PO_4_^3−^.” However, table columns and figure axes in that work were labelled only as “mg L^−1^” for both reactive P and total P. For added clarity in the future, we will explicitly include the full unit “mg L^−1^ PO_4_^3−^” or “mg L^−1^ P” in all tables and figure axes, as demonstrated in the present work.

From our experiences described here, particularly those during collaboration with a third-party laboratory, we now fully appreciate how critical it is for groups working on wastewater treatment technologies to report the correct P form in clear, specific units. Our concern that inconsistencies in P reporting are widespread is further supported by a recent systematic review of the literature on nutrient removal and recovery technologies by Kogler et al.^30^ In sorting reports for P removal efficiencies and effluent concentrations, three separate categories were required for reports of “TP = total phosphorus” and “PO_4_ = phosphate,” and “P = general phosphorus species if species was not identified clearly.”^30^ This review shows that there is a prevalent need to standardize quantitative performance metrics of nutrient removal and reuse technologies.

The ambiguity in reporting units introduces lingering questions of whether variable P concentrations in wastewaters across different studies is due to variability in the wastewater itself or variability in reporting and analysis methods. For example, Cid et al.^2^ reported 0.64 mmol L^−1^ “PO_4_^3−^ + HPO_4_^2−^” in average macerated toilet wastewater from one of their self-contained bathroom + wastewater treatment and recycling prototypes. The specific analytical method used in that work is not reported, but assuming based on the units that this represents a measurement of orthophosphate (reactive P), this would be approximately 60.8 mg L^−1^ PO_4_^3−^. This value is similar to what we report here for total P concentrations in macerated toilet wastewater, but also aligns well with our previous report of reactive P in toilet effluent.^14^ Another recent study by Reynaert et al.^27^ reported 36.2 mg L^−1^ average soluble phosphate “PO_4_” concentration in toilet wastewater influent from a urine-diverting toilet with solids separated from the flush water. While the use of the “PO_4_” unit is ambiguous, the analytical test used (Spectroquant) appears to give results in units of PO_4_-P. This would indicate that the average influent concentration in this study was 36.2 mg L^−1^ PO_4_-P or 111 mg L^−1^ PO_4_^3−^, and the nominal average concentration in the treated clean water tank is 25.4 mg L^−1^ PO_4_-P or 77.9 mg L^−1^ PO_4_^3−^,^27^ which would be unable to meet water reuse or discharge standards with nominal effluent P concentration limits (eg, <1 mg L^−1^ P in India).^36^

### Pitfall prevention: Preparation of mixed-P standard solutions for analytical test validation

For pitfall #1, we determined that validating test protocols and reporting units for reactive P, both in-house and with the third-party lab, can be accomplished by testing commercially-available standard solutions of orthophosphate. But what about validating methods when samples contain condensed and/or organic forms of P? Researchers should clarify the analytical methods used by explicitly stating whether samples were digested prior to analysis. For example, Chapter 4500-P in the 22nd edition of Standard Methods for the Examination of Water and Wastewater contains ten subsections detailing sample filtration pretreatments, three digestion methods, and three colorimetric methods.^32^ Therefore, simply referencing “standard method 4500-P” is insufficient for determining the fraction or form of P that has been analyzed. For samples containing condensed and/or organic P, definitive confirmation that digestion has been performed requires an analytical validation step testing a standard solution of known composition. However, the wastewater standard solutions readily available for purchase only contain orthophosphate. Testing these standard solutions will not clarify whether sample digestion is performed as required to convert all P forms to orthophosphate.

**Figure 3.**
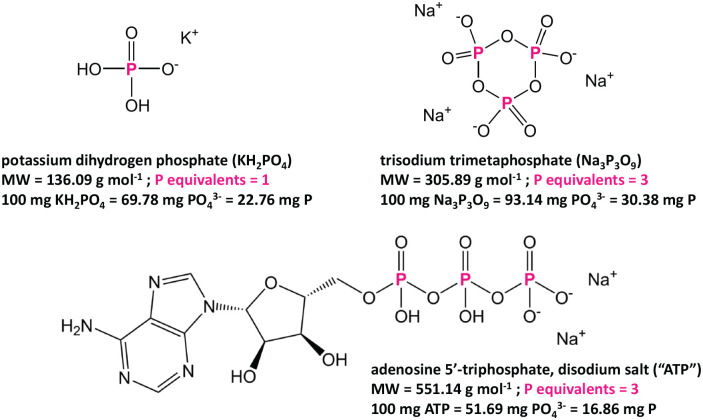
Chemical compounds used in this study to make in-house mixed-P standard solutions. KH_2_PO_4_ dissociates readily to give phosphate anions that are detected in reactive P tests. Na_3_P_3_O_9_ and ATP each contribute 3 phosphate equivalents, but these molecules must undergo acid digestion to release phosphate anions. Na_3_P_3_O_9_ and ATP are thus detected in total P tests but are not detected in reactive P tests.

We therefore set out to prepare our own in-house, mixed standard solution for validating total P testing protocols. In addition to KH_2_PO_4_ to provide orthophosphate, we identified two non-hazardous compounds which are readily-available and relatively inexpensive to represent condensed and organic P forms: trisodium trimetaphosphate (Na_3_P_3_O_9_) and adenosine 5’-triphosphate, disodium salt (ATP). Figure 3 shows the structures of these compounds and relevant properties, such as number of PO_4_^3−^ equivalents (i.e., number of PO_4_^3−^ anions detected in total P test per molecule of the compound). While KH_2_PO_4_ will be detected in a reactive P test, Na_3_P_3_O_9_ and ATP will only be detected in a total P test, as digestion is required to hydrolyze P-O and O-C bonds to release free orthophosphate into the solution.

**Figure 4.**
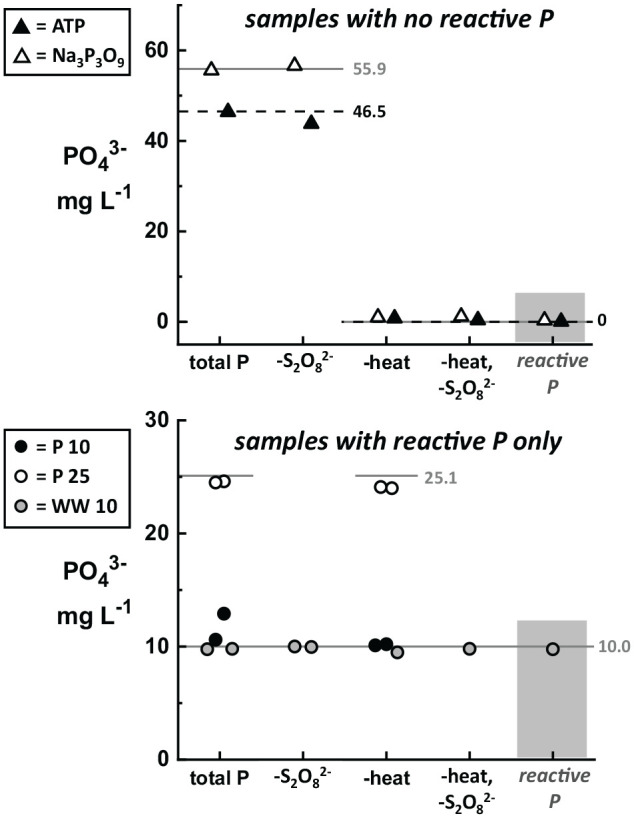
Demonstration of the different results obtained from total P and reactive P tests for identical samples, including tests where steps were systematically omitted from the total P protocol. Expected values are shown as horizontal reference lines (dashed lines = ATP). As expected, samples containing condensed and organic P (top, triangles) show no response to the reactive P test (shaded column). Samples containing ATP (closed symbols) and Na_3_P_3_O_9_ (open symbols) are accurately measured only when the total P test has been performed according to the protocol. Eliminating the heating step (“-heat” and “-heat, -S_2_O_8_^2−^”) results in a complete failure of the total P test. Eliminating only the persulfate pouch (“-S_2_O_8_^−2^”) shows only a small effect on the total P result for ATP. In contrast, samples that only contain reactive P (bottom, circles; WW 10, shaded symbols; P 25, open symbols; P 10, closed symbols) show nearly identical results for total P and reactive P (shaded column), and omitting steps from the total P protocol imparts no effect.

Solutions were first prepared containing only KH_2_PO_4_, Na_3_P_3_O_9_, or ATP and analyzed using total P and reactive P Hach kits (see Supplemental Material for a complete table of all solution recipes). Figure 4 shows these results by comparing samples that contain no reactive P (top; ATP only and Na_3_P_2_O_9_ only) to samples with only reactive P (bottom; P 25, P 10, WW 10). For samples with no reactive P (Na_3_P_3_O_9_ and ATP), the total P results match the expected values only when the heating step was included in the protocol. When heating was omitted from the protocol, both samples showed <1.5 mg L^−1^ PO_4_^3−^ for the total P measurement. As expected, the reactive P test for both Na_3_P_3_O_9_ and ATP measured almost zero (Na_3_P_3_O_9_ = 0.34 mg L^−1^ PO_4_^3−^; ATP = 0.08 mg L^−1^ PO_4_^3−^). Standard solutions purchased (WW 10) and prepared in-house (P 25, P 10) which only contained reactive P showed no difference in the values for the total P test when steps were omitted from the protocol. As expected, the results of the reactive P test and total P test are identical for samples containing only reactive P.

**Figure 5.**
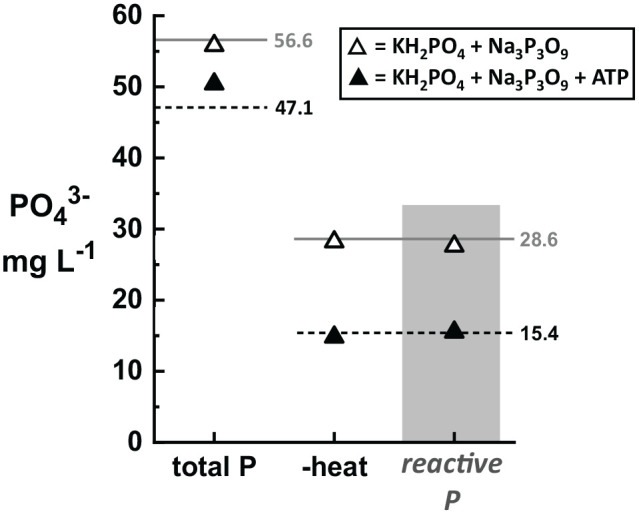
Comparison of total P and reactive P (shaded column) results for two mixed standard solutions prepared in-house. The 2-part mixed standard solution (open symbols) is a mixture of KH_2_PO_4_ and Na_3_P_3_O_9_. The 3-part mixed standard solution (closed symbols) contains KH_2_PO_4_, Na_3_P_3_O_9_, and ATP. Expected values are shown as horizontal lines (solid lines for 2-part standard solution; dashed lines for 3-part standard solution). Measured results for both mixed standard solutions were close to the expected values. When the heating step is omitted from the total P protocol, the measured value is the same as that for the reactive P test.

We also prepared two standard solutions with mixed-P forms that could be used to validate results from reactive P or total P tests using the same solution. Figure 5 shows total P and reactive P results for a 2-part standard solution (containing KH_2_PO_4_ and Na_2_P_3_O_9_) and a 3-part standard solution (containing KH_2_PO_4_, Na_3_P_3_O_9_, and ATP). Both the 2-part and 3-part standard solutions give measured total P concentrations close to their expected values. When the heating step is omitted, the total P results are nearly identical to the reactive P results.

The results in Figure 5 show that a 2-part standard solution of KH_2_PO_4_ and Na_3_P_3_O_9_ is sufficient to conclude whether the digestion heating step proceeds as necessary. These chemicals are affordable and do not require any special handling or storage considerations, so a solution of these two compounds could serve as a simple, yet robust mixed-P standard solution. The ATP salt has some disadvantages compared to Na_3_P_3_O_9_; it is more expensive, requires storage under refrigeration, and can have a variable water content bottle-to-bottle, making the calculation of the expected concentration slightly more complicated (see Supplemental Material).

However, there are two advantages for why some researchers may choose to include ATP in their in-house standard-solution formulation. First, as persulfate is expected to affect hydrolysis of organic P (ATP) but is not necessary for hydrolysis of condensed P (Na_3_P_3_O_9_), we hypothesized that including ATP in the mixture could validate that the persulfate reagent is included in the total P protocol. When the S_2_O_8_^2−^ is omitted from the solution of only Na_3_P_3_O_9_ or from the WW 10 standard solution (see Figure 4), the total P result does not decrease. In fact, there is a slight increase in the measured total P values (55.6 to 56.6 mg L^−1^ PO_4_^3−^ for Na_3_P_3_O_9_ from 9.8 to 10.0 mg L^−1^ PO_4_^3−^ for WW 10), although this change is well within the test-to-test variability demonstrated in Figure 1. On the other hand, when S_2_O_8_^2−^ is omitted from the solution of only ATP, the total P value measured decreases from 46.4 to 43.8 mg L^−1^. Similarly, for the 3-part mixture, omitting S_2_O_8_^2−^ gives a small decrease in total P from 50.4 to 49.4 mg L^−1^, however, this is on the border of the test-to-test variability of 1.0 to 1.5 mg L^−1^ shown in Figure 1. If validating inclusion of persulfate is desired, increasing the ratio of ATP in the 3-part mixed standard solution may be prudent. Alternatively, a 2-part standard solution containing only KH_2_PO_4_ and ATP would also be suitable. The second advantage of including ATP is that it also contains N atoms (5 N equivalents per ATP molecule) and can therefore be used to validate total nitrogen digestion protocols as well. Using the Hach low range total N kit (method 10071), we measured total N concentrations of 8.9 mg L^−1^ N in the ATP only solution and 2.0 mg L^−1^ N in the 3-part standard solution, both of which are in close agreement with the expected values of 11.4 and 2.3 mg L^−1^ N, respectively.

**Figure 6.**
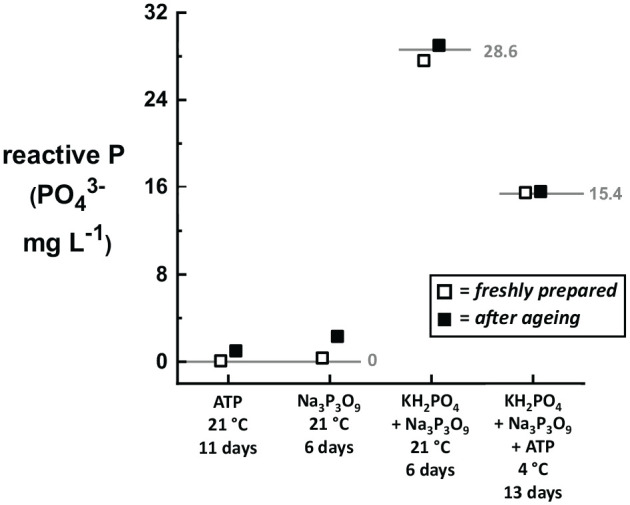
Comparison of reactive P results for freshly prepared samples (open symbols) and the same samples after ageing for several days (closed symbols) under laboratory conditions. Samples containing ATP and/or Na_3_P_3_O_9_ show very small increases in reactive P when stored at 20 °C for 6 to 11 days. The 3-part standard solution showed no increase in reactive P after 13 days when stored at 4 °C.

Finally, we evaluated the stability of the in-house mixed-P standard solutions over time, with and without refrigeration of the solutions. If either the Na_3_P_3_O_9_ or ATP is susceptible to degradation over time (e.g., slow hydrolysis), then the reactive P concentration of the standard solutions should increase as the samples age. Figure 6 shows reactive P values measured for Na_3_P_3_O_9_, ATP, 2-part, and 3-part mixtures after ageing for several days in sealed glass bottles. Only the 3-part mixture was stored under refrigeration; the other solutions were left in the laboratory ambient (21 °C). Small increases in the reactive P concentration were observed for all three solutions stored at room temperature (0.91, 1.98, and 1.40 mg L^−1^ PO_4_^3−^ for ATP, Na_3_P_3_O_9_, and the 2-part mixture, respectively) for 6 to 11 days. The 3-part mixture stored for 13 days at 4 °C increased by only 0.2 mg L^−1^ PO_4_^3−^. All of these values are within the test-to-test variability of 1.0 to 1.5 mg L^−1^ PO_4_^3−^, except for the Na_3_P_3_O_9_ solution, which is only slightly higher.

In summary, solutions prepared in-house with Na_3_P_3_O_9_ and/or ATP are sufficiently stable for several days without refrigeration to serve as standards for validation of total P protocols. These compounds show minimal degradation once dissolved in water and stored at room temperature. The mixed standard solutions can be used in-house or sent to third-party laboratories to confirm that the total P sample digestion protocol is followed correctly.

## Conclusions

Accurate and clear reporting of P in effluent from novel wastewater treatment technologies is critical for preventing eutrophication of receiving water bodies and in protecting human health. New research into methods to mitigate nutrient discharge in effluent from non-sewered sanitation systems and the adoption of nutrient discharge standards has many research groups stepping into new territory with phosphorus measurements. We have shown that commercially-available kits are accurate tools for evaluating reactive P and total P concentrations in standard solutions, and results were consistent across the two testing methods used in-house and at third-party lab once the correct unit conversion was applied. We also described options for making a standard solution containing condensed and/or organic P compounds that can be used to validate the digestion step in total P assays. We hope that the mixed-P standard solution recipes will be useful for other research groups, as commercially-available wastewater standard solutions only contain orthophosphate.

The work described here leads us to give recommendations for best practices going forward:

1) Regularly include P standard solutions of known concentration in P analyses. We advise that both an orthophosphate and organic phosphate standard solution (or a mixture) be used to validate which P assay is being used, determine the presence of a digestion step, and confirm the reported units.
2) Clearly state the fraction of phosphorus measured—reactive P, acid-hydrolyzable P, or total P—when reporting phosphorus results. Neither the units for reporting P nor a reference to “standard method 4500-P” are sufficient to convey this important information.
3) Discontinue use of the unit “PO_4_-P” in reporting results, and instead only use either PO_4_^3−^ or P. We think this may be the root of much confusion in the field, as (1) “PO_4_-P” and “P” units are interchangeable and therefore redundant, (2) “PO_4_-P” and “PO_4_^3−^” are *not* interchangeable, yet look quite similar, and (3) “PO_4_-P” is not a chemically meaningful notation (see Supplemental Material for further discussion).

We hope that these recommendations will be useful and widely adopted by groups (including ours) who are adding or continuing with total P analysis as part of their routine testing.

## Supplemental Material

sj-pdf-1-ehi-10.1177_11786302211019218 – Supplemental material for Potential Pitfalls in Wastewater Phosphorus Analysis and How to Avoid Them Click here for additional data file. Supplemental material, sj-pdf-1-ehi-10.1177_11786302211019218 for Potential Pitfalls in Wastewater Phosphorus Analysis and How to Avoid Them by Praveen Rosario, Ramya Viswash, Thamayanthi Seenivasan, Sudha Ramalingam, Katelyn L Sellgren, Sonia Grego and Lena Trotochaud in Environmental Health Insights
