# Supplementary material for: Potential Pitfalls in Wastewater Phosphorus Analysis and How to Avoid Them
Source: Environ Health Insights. 2021 May 30;15:11786302211019218. doi: 10.1177/11786302211019218 (PMC8168049; doi:10.1177/11786302211019218)
Supplement: sj-pdf-1-ehi-10.1177_11786302211019218 – Supplemental material for Potential Pitfalls in Wastewater Phosphorus Analysis and How to Avoid Them [file sj-pdf-1-ehi-10.1177_11786302211019218.pdf]

Supplemental Material for:

Potential pitfalls in wastewater phosphorus analysis and how to avoid them

Praveen Rosario,[1] Ramya Viswash,[2] Thamayanthi Seenivasan,[2]
Sudha Ramalingam,[2] Katelyn L. Sellgren,[3] Sonia Grego,[3] Lena Trotochaud[3]*

[1] RTI International India, New Delhi, India
[2] PSG Institute for Medical Science and Research, Coimbatore, TN, India
[3] Duke University Center for Water, Sanitation, Hygiene, and Infectious Disease (WaSH-AID) and Department of Electrical and Computer Engineering, Durham, NC, USA
* lena.trotochaud@duke.edu

Contents:

- **Table S1.** Characteristics of the commercially-available wastewater standards (WW 10 and WW 30).
- **Table S2.** Characteristics of the salts used to prepare in-house mixed-P standards.
- **Table S3.** Recipes used to prepare in-house standard solutions.
- Detailed step-by-step procedures of the Hach methods for reactive P (8048) and total P (8190).
- Example stoichiometric calculations for determining expected PO_4^{3-} concentrations in standard solutions prepared in-house.
- Further discussion on the preference for chemically meaningful notation in units for reporting phosphorus.

Table S1. Characteristics of the commercially-available wastewater standards (WW 10 and WW 30).

HACH product number	LCA720	2833149
volume (mL)	250	500
price in USD (as of Feb. 2021)	\$46.95	\$101.00
wastewater standard name (from manufacturer)	mixed-parameter standard, NIST, wastewater, high range	wastewater influent inorganics quality control standard
**wastewater standard abbreviation (used in this work)**	**WW 30**	**WW 10**
**P units**	**PO_4-P**	**PO_4^{3-}**
**P content (mg L^{-1} in units given above)**	**10.0**	**10**
COD (mg L^{-1})	500	500
NH_3-N (mg L^{-1})	25.0	15
NO_3-N (mg L^{-1})	25.0	10
total N (mg L^{-1})*	50.0	25
SO_4^{2-} (mg L^{-1})	--	400
TOC (mg L^{-1})	200	161

*Total N concentration is the sum of NH_3-N and NO_3-N concentrations.

Table S2. Characteristics of the salts used to prepare in-house mixed-P standards.

Sigma-Aldrich product number	P5655-500G	T5508-500G	A26209-1G
quantity (g)	500	500	1
price in USD (as of Feb. 2021)	\$70.90	\$35.40	\$43.30
price per g	\$0.14	\$0.07	\$43.30
CAS number	7778-77-0	7785-84-4	34369-07-8
chemical name	potassium dihydrogen phosphate	trisodium trimetaphosphate	adenosine 5'-triphosphate disodium salt (**ATP**)
chemical formula	**KH_2PO_4**	**$Na_3P_3O_9$**	$Na_2C_{10}H_{14}N_5P_3O_{13} \cdot xH_2O$
molecular weight (g $mole^{-1}$)	136.09	305.89	551.14 [a]
lot number	SLBS0199V	SLCD1552	MKCM6750
lot purity	100.0%	100%	90% [b]
storage temperature	"room temp"	none listed	2 – 8 °C

[a] Molecular weight does not include water molecules.

[b] Purity listed in the table accounts for 9.0% water and 1.0% solvent of crystallization, as indicated in the Certificate of Analysis.

Table S3. Recipes used to prepare in-house standard solutions.

standard solution	volume (L)	mass KH_2PO_4 (g)	mass $Na_3P_3O_9$ (g)	mass ATP (g)	reactive P expected (mg L^{-1} PO_4^{3-})	total P expected (mg L^{-1} PO_4^{3-})
P 10	1 *	1.44	--	--	10.0	10.0
P 25	1 **	0.36	--	--	25.1	25.1
$Na_3P_3O_9$	0.5	--	0.030	--	0	55.9
ATP	0.5	--	--	0.050	0	46.5
2-part	1	0.041	0.030	--	28.6	56.6
3-part	0.5	0.011	0.012	0.010	15.4	47.1

* This recipe gives 1005 mg L^{-1} PO_4^{3-} and must be further diluted 100× to give 10.0 mg L^{-1}.
** This recipe gives 251 mg L^{-1} PO_4^{3-} and must be further diluted 10× to give 25.1 mg L^{-1}.

Detailed step-by-step procedures of the Hach methods for reactive P (8048) and total P (8190).

Total P (method 8190)

The "Test 'N Tube" low range kits (Hach product number 2742645) were used for this method at both the India field-testing site and the US lab at Duke. The detection range for this test kit is 0.06 – 3.50 mg L^{-1} PO_4^{3-}. The sensitivity of the method listed by the manufacturer is 0.06 mg L^{-1} PO_4^{3-} per 0.010 change in absorbance.

- 5 mL of sample (or sample diluted with water, as necessary) is added to a glass reaction vial.
- The contents of one potassium persulfate powder pillow are added to the sample vial. The the vial is inverted/shaken repeatedly to dissolve the powder.
- The sample vial is digested at 150 °C for 30 min using a heating block (Hach, DRB 200).
- The sample is removed from the heating block and cooled to room temperature.
- 2 mL of 1.54 N NaOH is added to the vial. The vial is inverted to mix and wiped clean.
- This sample vial is used to zero the colorimeter (program 536 P Total/AH PV TNT), i.e. the sample is measured as the blank.
- The contents of one PhosVer 3 powder pillow are added to the vial. The vial is shaken for 20-30 s (the powder does not dissolve completely) and allowed to react for 2-8 min before reading the final result.

Reactive P (method 8048)

Two kits are available from Hach for measuring reactive P method 8048. The "Test 'N Tube" low range kit (Hach product number 2742545) was used in the US lab at Duke; the detection range for this test kit is 0.06 – 5.00 mg L^{-1} PO_4^{3-}. The sensitivity of the method listed by the manufacturer is 0.06 mg L^{-1} PO_4^{3-} per 0.010 change in absorbance. Alternatively, the PhosVer3 Powder Pillows (Hach product number 2106046) can also be purchased on their own for use with a reusable glass sample cell (Hach product number 2401906); this latter option was used at the

India field-testing site and has a detection range of 0.02 – 2.50 mg L^{-1} PO_4^{3-}. The sensitivity of this method listed by the manufacturer is 0.02 mg L^{-1} PO_4^{3-} per 0.010 change in absorbance.

For the "Test 'N Tube" low range kit:

- 5 mL of sample (or sample diluted with water, as necessary) is added to a glass reaction vial, and the vial is inverted several times to mix.
- The sample vial is wiped clean and used to zero the colorimeter (program 535 P React. PV TNT), i.e. it is measured as the blank.
- The contents of one PhosVer3 powder pillow are added to the vial, the vial is shaken for 20-30 s, and allowed to react for 2 min before reading the final result.

For the Powder Pillows used with the reusable sample cell:

- 10 mL of sample (or sample diluted with water, as necessary) is added to the reusable glass sample cell.
- The sample cell is wiped clean and used to zero the colorimeter (program 490 P React. PP), i.e. it is measured as the blank.
- The contents of one PhosVer3 powder pillow are added to the sample cell, the cell is capped and shaken for 20-30 s, and allowed to react for 2 min before reading the final result.

Example stoichiometric calculations for PO_4^{3-} concentration in standard solutions prepared in-house.

Solutions of KH_2PO_4 will contain reactive phosphate. This salt will be measured in both reactive P and total P tests. For solutions containing $Na_3P_3O_9$ or ATP, these salts will only contribute to the measurement of the total P tests. For the example stoichiometric calculations below, we will use the recipe for the 3-part standard given above in Table S3.

To determine the expected phosphate concentration, we first calculate the mass in mg of phosphate in the mass of salt weighed from the reagent bottle. The molecular weight of the salt and the lot purity (see Table S2) are important for this calculation, as well as the number of P equivalents in the compound (see Figure 3 in the main text).

For the KH_2PO_4 (contribution to both reactive P and total P tests):

$$?\,\text{mg}\,PO_4^{3-} = \frac{0.011\text{ g salt}}{} \times \frac{1000\text{ mg salt}}{1\text{ g salt}} \times \overbrace{\frac{100\text{ mg }KH_2PO_4}{100\text{ mg salt}}}^{\text{lot purity}} \times \frac{(\overbrace{1\,\times}^{\text{\# of P equivalents}} 94.97\text{ mg }PO_4^{3-})}{\underbrace{136.09\text{ mg }KH_2PO_4}_{\text{molecular weight}}} = 7.68\text{ mg }PO_4^{3-}$$

Next we divide by the volume of water the salt is dissolved in to get the concentration:

$$? \text{ mg L}^{-1}\ \text{PO}_4^{3-} = \frac{7.68 \text{ mg PO}_4^{3-}}{\boxed{0.5 \text{ L}}} = 15.4 \text{ mg L}^{-1}\ \text{PO}_4^{3-}$$

solution volume

Similar calculations are used for solutions of $Na_3P_3O_9$ or ATP. The molecular weights, lot purity, # of P equivalents, and solution volume must be adjusted accordingly.

For the $Na_3P_3O_9$ (contribution only to total P test):

lot purity | # of P equivalents

$$? \text{ mg PO}_4^{3-} = \frac{0.012 \text{ g salt}}{} \times \frac{1000 \text{ mg salt}}{1 \text{ g salt}} \times \boxed{\frac{100 \text{ mg Na}_3\text{P}_3\text{O}_9}{100 \text{ mg salt}}} \times \frac{(\boxed{3\ \times} 94.97 \text{ mg PO}_4^{3-})}{\boxed{305.89 \text{ mg Na}_3\text{P}_3\text{O}_9}} = 11.2 \text{ mg PO}_4^{3-}$$

molecular weight

$$? \text{ mg L}^{-1}\ \text{PO}_4^{3-} = \frac{11.2 \text{ mg PO}_4^{3-}}{\boxed{0.5 \text{ L}}} = 22.4 \text{ mg L}^{-1}\ \text{PO}_4^{3-}$$

solution volume

For the ATP (contribution only to total P test):

lot purity | # of P equivalents

$$? \text{ mg PO}_4^{3-} = \frac{0.010 \text{ g salt}}{} \times \frac{1000 \text{ mg salt}}{1 \text{ g salt}} \times \boxed{\frac{90 \text{ mg ATP}}{100 \text{ mg salt}}} \times \frac{(\boxed{3\ \times} 94.97 \text{ mg PO}_4^{3-})}{\boxed{551.14 \text{ mg ATP}}} = 4.65 \text{ mg PO}_4^{3-}$$

molecular weight

$$? \text{ mg L}^{-1}\ \text{PO}_4^{3-} = \frac{4.65 \text{ mg PO}_4^{3-}}{\boxed{0.5 \text{ L}}} = 9.31 \text{ mg L}^{-1}\ \text{PO}_4^{3-}$$

solution volume

The expected value of reactive P (15.4 mg L^{-1} PO_4^{3-}) for the 3-part standard is the same as the concentration of PO_4^{3-} from KH_2PO_4, as this is the only compound which contributes to reactive P.

The expected value of total P (47.1 mg L^{-1} PO_4^{3-}) is the sum of the contributions from KH_3PO_4, $Na_3P_3O_9$, and ATP: 15.4 + 22.4 + 9.31 = 47.1 mg L^{-1}.

Further discussion on the preference for chemically meaningful notation in units for reporting phosphorus.

We have found that in wastewater treatment analyses, the unit "P" is used equivalently and often interchangeably to "PO_4-P". This is technically correct, as only the P atoms are counted in both cases. However, we think this may be the root of much confusion in the field, as (1) "PO_4-P" and "PO_4^{3-}" are *not* interchangeable, yet look quite similar, and (2) "PO_4-P" is not a chemically meaningful notation.

The notation "PO_4^{3-}" for orthophosphate has chemical meaning; this is the correct chemical notation for the phosphate ion and both P and O atoms are counted when reporting in mg L^{-1} PO_4^{3-}. Similarly, using "P" for "total phosphorus" is chemically intuitive, as P is the elemental symbol for phosphorus and only the P atoms are counted. However, "PO_4-P" is essentially a shorthand notation for the spoken phrase "orthophosphate as phosphorus", but does not follow the International Union of Pure and Applied Chemistry (IUPAC) conventions for chemical notations. The use of a hyphen or dash in a chemical formula indicates the presence of a chemical bond, which does not make sense in this notation, as there is not a second P atom bound to a PO_4 fragment; i.e. the second "P" in "PO_4-P" is redundant from a chemistry perspective. Additionally, only the mass of the P atom is counted when reporting in PO_4-P units, so including the "O_4" atoms in the unit notation is confusing.

We therefore recommend to discontinue use of "PO_4-P" when reporting wastewater analyses. Continuing to utilize two redundant units (PO_4-P and P) to report the same information provides no benefit and introduces potential sources of confusion.
